# Elemental analysis of lung tissue particles and intracellular iron content of alveolar macrophages in pulmonary alveolar proteinosis

**DOI:** 10.1186/1465-9921-12-88

**Published:** 2011-06-30

**Authors:** Yasuo Shimizu, Shinichi Matsuzaki, Kunio Dobashi, Noriko Yanagitani, Takahiro Satoh, Masashi Koka, Akihito Yokoyama, Takeru Ohkubo, Yasuyuki Ishii, Tomihiro Kamiya, Masatomo Mori

**Affiliations:** 1 Department of Medicine and Molecular Science, Gunma University Graduate School of Medicine, 3-39-15 Showa-machi, Maebashi, Gunma 371-8511, Japan; 2 Department of Pulmonary Medicine, Maebashi Red Cross Hospital 3-21-36 Asahi-cho Maebashi, Gunma 371-0014, Japan; 3 Gunma University Faculty of Health Science, 3-39-15 Showa-machi, Maebashi, Gunma 371-8511, Japan; 4 Japan Atomic Energy Agency, Takasaki Advanced Radiation Research Institute, 1233, Watanuki-machi, Takasaki, Gunma 370-1292, Japan

## Abstract

**Background:**

Pulmonary alveolar proteinosis (PAP) is a rare disease occurred by idiopathic (autoimmune) or secondary to particle inhalation. The in-air microparticle induced X-ray emission (in-air micro-PIXE) system performs elemental analysis of materials by irradiation with a proton microbeam, and allows visualization of the spatial distribution and quantitation of various elements with very low background noise. The aim of this study was to assess the secondary PAP due to inhalation of harmful particles by employing in-air micro-PIXE analysis for particles and intracellular iron in parafin-embedded lung tissue specimens obtained from a PAP patient comparing with normal lung tissue from a non-PAP patient. The iron inside alveolar macrophages was stained with Berlin blue, and its distribution was compared with that on micro-PIXE images.

**Results:**

The elements composing particles and their locations in the PAP specimens could be identified by in-air micro-PIXE analysis, with magnesium (Mg), aluminum (Al), silicon (Si), phosphorus (P), sulfur (S), scandium (Sc), potassium (K), calcium (Ca), titanium (Ti), chromium (Cr), copper (Cu), manganase (Mn), iron (Fe), and zinc (Zn) being detected. Si was the major component of the particles. Serial sections stained by Berlin blue revealed accumulation of sideromacrophages that had phagocytosed the particles. The intracellular iron content of alveolar macrophage from the surfactant-rich area in PAP was higher than normal lung tissue in control lung by both in-air micro-PIXE analysis and Berlin blue staining.

**Conclusion:**

The present study demonstrated the efficacy of in-air micro-PIXE for analyzing the distribution and composition of lung particles. The intracellular iron content of single cells was determined by simultaneous two-dimensional and elemental analysis of paraffin-embedded lung tissue sections. The results suggest that secondary PAP is associated with exposure to inhaled particles and accumulation of iron in alveolar macrophages.

## Background

Pulmonary alveolar proteinosis is a rare disease characterized by dense accumulation of surfactant and phospholipids in the alveoli and distal airways [1]. Progression of this disease leads to respiratory failure [2]. Auto anti-granulocyte-macrophage colony-stimulating factor (anti-GM-CSF) antibody is involved in the development of the idiopathic (autoimmune) form of PAP [3]. PAP may also associate with malignancies and secondary to particle exposures [4-8]. Considering the latter, a recent report from Japan revealed exposure to dust in 23% of 223 cases of PAP [9]. Thus, particles are considered to be one of the causative agents of secondary PAP. Disturbance of iron (Fe) homeostasis has been reported in idiopathic PAP patients. Present knowledge provides little information about the mechanisms behind the observed accumulation of iron in lung tissues and alveolar macrophages. However, in cases of secondary PAP, Fe bound to the inhaled particles may be a potential source of iron [10,11]. Also, Fe-catalyzed oxidant-induced rupture of lysosomes and consequent apoptosis of alveolar macrophages has been proposed to be involved in idiopathic PAP. To follow disease progression, routine examination for haemosiderin (Fe) in the macrophages of idiopathic PAP patients has been proposed [11].

The aim of this study was to assess the secondary PAP due to inhalation of harmful particles by employing in-air microparticle induced X-ray emission (in-air micro-PIXE) analysis for particles and intracellular iron in lung tissue specimens combined with Berlin blue staining for iron.

## Methods

### Patient and sample preparation

PAP lung tissue was obtained from a 64-year-old woman at video-assisted thoracoscopic surgery (VATS). She was a hairdresser, and a current smoker (10 pack-years). Serum anti-GM-CSF antibody was negative analysis. Pathological examination revealed interstitial pneumonia with interstitial fibrosis and periodic acid-Schiff (PAS)-positive material in the alveolar spaces. The pathological diagnosis was pulmonary alveolar proteinosis. As a control, normal lung tissue was obtained from a 72-years-old woman with lung cancer of adenocarcinoma. She was a housewife, and a never smoker without history of occupational exposure of particles. She received a lobectomy at surgical resection, and the normal lung of the margin of tumor was used for the analysis. Tissues were subjected to in-air micro-PIXE analysis and Berlin blue staining for iron.

### In-air micro-PIXE analysis

**Figure 1:**
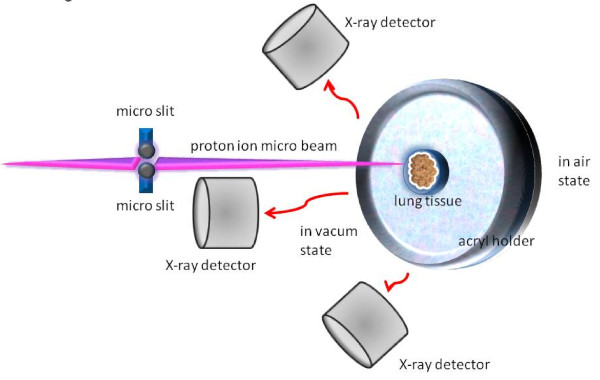
**In-air micro-PIXE system**. The proton ionmicrobeam from the accelerator is focused through microslit, and the beam is irradiated to the tissue sample in vacum state. The characteristic X-rays, those are specific energy for each element produced by irradiation, are identified by the X-ray detectors.

For in-air micro-PIXE analysis, paraffin-embedded lung tissue specimens were cut into sections 5 μm thick. Each section was dried, placed onto 5 μm polycarbonate film, and fixed in the sample holder as described previously [12]. After irradiation with a 3.0 MeV proton beam, a microbeam was extracted for micro-PIXE analysis of the characteristic X-ray patterns of various elements (Figure 1). The elemental map of phosphorus (P) was used to identify the shape of the cells, and sulfur (S) was used to demonstrate surfactant [13]. Iron (Fe) to P ratio was used for comparison of intracellular iron content [14]. Berlin blue staining was performed on serial sections adjacent to the micro-PIXE sections, and microscopy was done with a BH-4 (Olympus, Japan). The in-air micro-PIXE system was located at the TIARA facility of the Japan Atomic Energy Agency (JAEA). This study was conducted according to the guidelines of the Declaration of Helsinki, and it was approved by the Human Research Committee of Gunma University.

## Results

### In-air micro-PIXE analysis of dense particles area in PAP tissue

**Figure 2:**
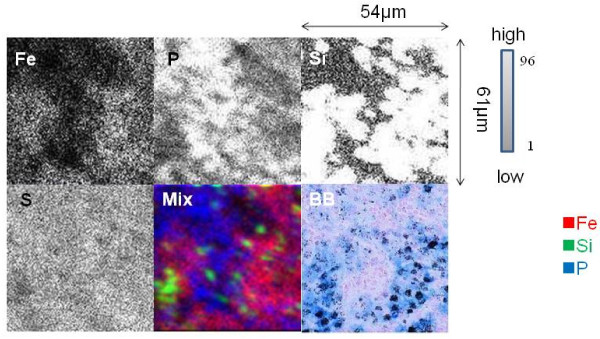
**In-air micro-PIXE analysis of an area of dense particles phagocytosed by macrophages in lung tissue from the PAP patient**. The microbeam was focused on an area of 54 μm × 61 μm. Two-dimensional analysis was performed on the distribution and intensity of elements in the dense particle area of the lung. The strength of Fe, P, Si, and S in lung tissue is shown by gray to white dots. The Si content is high on the elemental map. The content and distribution of Fe, Si, and P is shown in mixed colors (Mix) as follows: Fe (red), Si (green), and P (blue). A serial section of the area subjected to micro-PIXE showed dense black particles and accumulation of macrophages by Berlin blue staining (BB) (×1000). Sideromacrophages containing rich iron (stained blue) phagocytosed the particles (black).

Berlin blue staining revealed that basically, two morphologic characteristics of present PAP case needed to study, i.e. in lung tissue cells with dense particles and alveolar macrophages in the alveoli digesting deposits of surfactant. Elemental analysis of the PAP lung tissue was performed on an area containing dense particles phagocytosed by macrophages (54 μm × 61 μm) with the focused beam. High Kα peaks of magnesium (Mg), aluminum (Al), silicon (Si), phosphorus (P), sulfur (S), scandium (Sc), potassium (K), calcium (Ca), titanium (Ti), chromium (Cr), copper (Cu), manganese (Mn), iron (Fe), and zinc (Zn) were obtained. The Kβ peak of Fe appeared separately from Kα peak, and near the peak of cobalt (Co) (data not shown). The elemental map showed a high Fe contents strongly associated with Si, as well as metals in the particles. Serial sections of lung tissue with Berlin blue staining showed dense black particles that had been phagocytosed and accumulated in iron-rich alveolar macrophages (Figure 2).

### In-air micro-PIXE analysis of alveolar macrophages in surfactant-rich area

**Figure 3:**
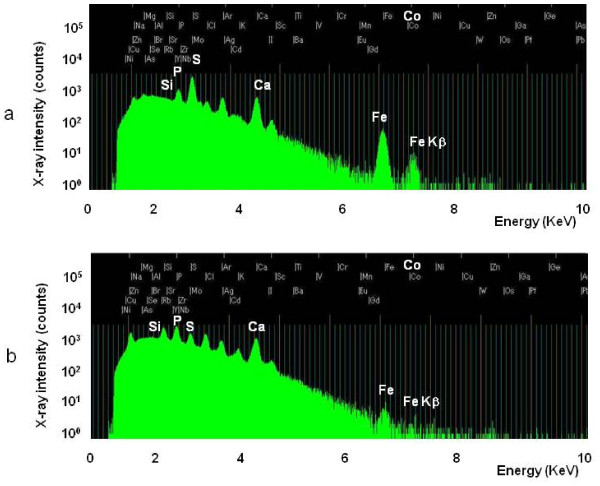
**The X-ray peaks for each element obtained by in-air micro-PIXE analysis of alveolar macrophages from the surfactant-rich area in PAP and control lung**. The microbeam was focused on a 54 μm × 61 μm area of the PAP lung tissue. Peaks display the characteristic X-ray signatures for each element, as shown by the counts (a). High peaks of S, Ca, and Fe were detected. The peak for Fe Kβ is near the peak of cobalt. The microbeam was focused on a 54 μm × 61 μm area of the control lung tissue (b). Peaks of S and Fe were lower than PAP lung tissue.

**Figure 4:**
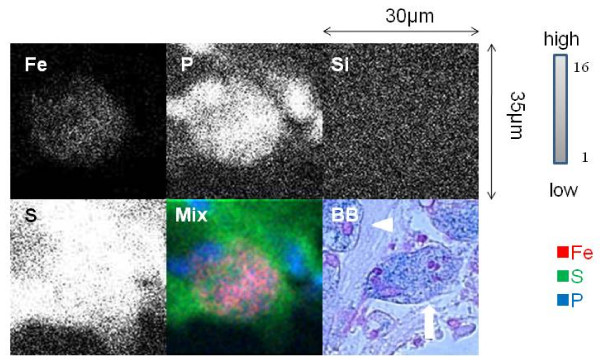
**In-air micro-PIXE analysis of an alveolar macrophage from the surfactant-rich area of PAP lung**. The microbeam was focused on a 30 μm × 35 μm area of the lung to analyze the intracellular distribution of elements in an alveolar macrophage. Two-dimensional analysis was performed on the intracellular distribution and intensity of elements in an alveolar macrophage. The strength of Fe, P, Si, and S in lung tissue is shown by gray to white dots. Cell morphology was identified by the distribution of P located in the surfactant-rich area, which was identified by the distribution of S. The intracellular content and distribution of Fe, S and P in an alveolar macrophage are shown in mixed colors (Mix) as follows: Fe (red), S (green), and P (blue). A serial section of the area subjected to micro-PIXE showed sideromacrophages (arrow) containing iron (blue) (× 1000) by Berlin blue staining (BB) in surfactant (arrowhead).

**Figure 5:**
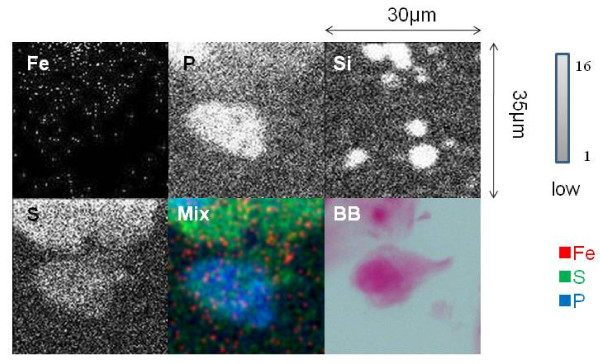
**In-air micro-PIXE analysis of an alveolar macrophage from the control lung**. The microbeam was focused on a 30 μm × 35 μm area of the control lung to analyze the intracellular distribution of elements in an alveolar macrophage. Two-dimensional analysis was performed on the intracellular distribution and intensity of elements in an alveolar macrophage. The strength of Fe, P, Si, and S in lung tissue is shown by gray to white dots. Cell morphology was identified by the distribution of P located in normal lung area. The intracellular content and distribution of Fe, S and P in an alveolar macrophage are shown in mixed colors (Mix) as follows: Fe (red), S (green), and P (blue) (d). A serial section of the area subjected to micro-PIXE showed a negative stained iron in a macrophage for Berlin blue (BB) (× 1000).

Elemental analysis of the alveolar macrophages from a surfactant-rich area (54 μm × 61 μm) with the focused beam area showed high S and Fe peaks (Figure 3a), however in the control lung tissue (54 μm × 61 μm) with the focused beam area, peaks of S and Fe were apparently lower than PAP lung tissue (Figure 3b). Elemental analysis of the PAP lung tissue was performed on an alveolar macrophage in the surfactant-rich area (30 μm × 35 μm) with the focused beam (Figure 4). The distribution of intracellular elements in a macrophage indicated accumulation of Fe, and this distribution was corresponded with the cell morphology indicated by P surronded by S-containing surfactant. Serial sections of lung tissue with Berlin blue staining showed iron-rich alveolar macrophages. In contrast, intracellular Fe in a macrophage of control lung was very low by in-air micro-PIXE analysis, and serial sections of lung tissue did not show iron staining in alveolar macrophages by Berlin blue staining (Figure 5). Silica particles were detected in the lung tissue structure.

### Quantitative analysis for iron in tissue section

The Fe/P ratios calculated by in-air PIXE analysis were 0.28, 0.36 and 0.0036 for a dense particles phagocytosed by macrophages in PAP, an alveolar macrophage in surfactant-rich area of PAP and an alveolar macrophage of control, respectively.

## Discussion

Disturbance of iron homeostasis has been reported in PAP [10], and alveolar macrophages from BAL have a high Fe content [11]. In that study, the cellular distribution of iron was evaluated by Berlin blue staining, and measurement of the cellular Fe content was done by atomic absorption spectrometry after lysis of the cells. In the present study, there are two morphologic characteristics of this PAP-case needed to study, the first in the lung tissue cells (mainly siderophages) with dense particles containing large amounts of Si and Fe, and the second in alveolar macrophages in the alveoli containing large amounts of iron in intracellulary digesting deposits of surfactant. In-air micro-PIXE system was used to assess the distribution of intracellular Fe in macrophages. The Fe/P ratio has been used for evaluation of iron overload to the cells [14]. Present study revealed that the Fe/P ratio in a single macrophage in PAP was very high compared to control lung. Silica particles were detected in control lung. Silica deposition is frequently observed in normal lung without history of occupational exposure [15]. In control lung, it seemed that silica particles did not increase intracellular iron of macrophages by analysis of in-air micro PIXE and Berlin blue staining. Elemental analysis showed the Kβ peak of Fe appeared separately from Kα peak, and near the peak of cobalt (Co). The Kα peak appears when an electron transits from L to K electron shell by irradiation for sample, and the Kβ peak appears when an electron transits from M to K electron shell by irradiation for sample. In our micro-PIXE system, the peaks of Kα and Kβ for light element appear close to each other because of nearly energy levels. However, the peaks of Kα and Kβ for heavy elements, in present case Fe, appear separately. In present case, the calculation of Fe/P ratio was performed using the formula taking account Kα for heavy elements, as previously [12,16].

Cases of PAP had been reported in association with occupational and environmental exposure to substances such as indium oxide, indium-tin oxide, silica, titanium, aluminum, cotton, and fibrous material [4-8]. A recent study from Japan showed that exposure to dust was associated with PAP [9]. In the present study, in-air-micro-PIXE analysis revealed the existence of particles with a high Si contents with Fe in lung tissue from a PAP patient. There has already been a report about a PAP patient who was a hairdresser [17], but the association between particles and the materials used by hairdressers could not be assessed in present case. Although the association of cigarette smoking and PAP has not been determined [9], tobacco smoke could not be excluded as the source of the iron. However, it is necessary to examine lung particles derived from smoking by in-air micro-PIXE in a setting with few environmental factors such as an animal model.

As a factor in the onset of PAP, iron-induced oxidative stress and lysosomal rupture following the disturbance of iron homeostasis may play a role [10,11]. In this study, the Fe/P ratio was measured in an alveolar macrophage from PAP lung tissue sections, while Berlin blue staining revealed an abundance of haemosiderin inside alveolar macrophages. In a previous study, a high Fe concentration was detected in alveolar macrophages isolated from the broncho-alveolar lavage fluid of PAP patients [10], and it was suggested that assessment of lysosomal iron (reflected by the number of haemosiderin-laden alveolar macrophages in bronchoalveolar lavage fluid) might serve as a marker of the progression and prognosis of PAP.

## Conclusions

Application of in-air micro-PIXE is possibly useful for evaluation of iron as a disease marker of PAP, assessing the distribution of iron in particles and alveolar macrophages, and for determining the intracellular iron content in alveolar macrophages. Secondary PAP is associated with exposure to inhaled particles and accumulation of iron in alveolar macrophages.

## Competing interests

The authors declare that they have no competing interests.

## Authors' contributions

YS designed this study, prepared the sample, immunostained the lung tissues, analysed the datas, and wrote this manuscript. SM prepared the sample, analysed the datas and irradiated to the sample. NY prepared the sample, TS analysed the datas, irradiated the sample and gave useful suggestion on this study. MK, AY, TO, YI, TK irradiated to the sammple. KD irradiated the sample and gave useful suggestions on this study. MM gave useful suggestion on this study.

## Acknoelwdgements

We thank Norio Horiguchi M.D, Gunma University and Hideaki Itoh M.D., Meabashi Red Cross Hospital for facilitation of microscopic analysis. This work was not supported by any grant. None of the authors declare competing financial interests.
